# The Hospital World

**Published:** 1911-03-25

**Authors:** 


### THE HOSPITAL WORLD.

### Personalia.

The four newly-appointed trustees of the Hospital Sunday Fund, Sir John Charles Bell, Sir Charles Ernest Tritton, Mr. John Hampton Hale, and Mr. John Henry Buxton, are men well known in the City and not new to the work of the Fund. It is hoped that their appointment, by doing away with the system of transfers, which in the old arrangement complicated very much the finances of the fund, will make its machinery more simple and efficient. Their status and training should be invaluable. Sir J. C. Bell, who was Lord Mayor during 1908, is managing director of the Wenlock Brewery Company, and Sir Ernest Tritton is the senior partner in Brightwen and Co., the banking agents well known in Lombard Street. All four gentlemen have been members of the Finance Committee of the Sunday Fund, and, as we need not remind our readers, Mr. John Hale, the underwriter, has been its chairman.

The resignation by Miss Mollett of the matroncy of the Royal South Hants and Southampton Hospital removes a very well-known personality from this institution. Miss Mollett's career has embraced the period during which most of the modern reforms in nursing have taken place, and the fact that she has remained till so recently as last week at the head of a great provincial hospital shows that from the first she was a progressive member of her profession, if indeed she did not initiate more than one of these reforms herself. Institutional workers everywhere will congratulate Miss Mollett on the retirement which her active career during many years has certainly earned. The assistant matron, Miss Winterscale, has also resigned.

The announcement which was recently made at the annual meeting of the Royal Free Hospital, of which a report appears in another column, that Mr. Conrad Thies is resigning the post of Secretary, which he has held for so many years, has come as a surprise to many. In response to an inquiry as to the reason for his resignation, Mr. Thies states that after nearly half a century of active business life he feels that he has earned the right to some leisure, and that it will probably prove beneficial to the Royal Free if he makes room for a more energetic man. He hopes, moreover, to be able to maintain his keen interest in the work of the Royal Free and in hospital matters generally. In a later issue we shall publish some particulars respecting Mr. Thies' past career, and the striking progress which the Royal Free Hospital has made under his direction. In the meantime all hospital workers will agree that Mr. Thies has deserved a rest, and will wish him every good fortune on the travels which it is expected he will undertake.

One of the members of the Committee of the General Hospital, Tunbridge Wells, Mr. W. Skinner, has shown for the past twenty-five years the extraordinary value of small sums subscribed to hospitals. In his place of business he has a box, in which he receives apparently insignificant sums from the "very poor," and by dint of persevering effort Mr. Skinner collects in this way on the average £20 per annum. His total contribution to the Hospital collected up to date is £500. Personal initiative has combined here with personal service to produce a most successful result.

The retirement of Sir Charles Whitehead from the chairmanship of the Kent County Ophthalmic Hospital after the wonderful period of thirty-four years is not the only loss which the hospital has felt recently. The secretary, Mr. Samuel Harman, who retired after nineteen years' service, is succeeded by his son, Mr. Coulter Harman, who begins his duties in a reorganised secretarial department, in a remodelled office, and who will give his whole time to the work. This, which has grown, of course, with the record number of attendances, is now put on up-to-date lines by being done entirely on the hospital premises, and many of the hospital friends will regret that the late Mr. Samuel Harman did not enjoy his retirement long enough to see the new development in full progress.

### Elections and Appointments.

Mr. J. B. Clarke has been elected a vice-president of the Birmingham General Hospital.

The Duke of Bedford has been elected President of University College Hospital.

Sir W. Crundall has been re-elected President of the Royal Victoria Hospital, Dover.

The Earl of Plymouth has been re-elected President of the Birmingham and Midland Eye Hospital.

Mr. C. S. Wollen has been re-elected hon. secretary, and Mr. I. F. Rockhey hon. treasurer of the Torbay Hospital.

Mr. Macleod Yearsley, F.R.C.S., has been appointed honorary consulting aural surgeon to the Royal School for Deaf and Dumb Children, Margate.

Mr. Goode, Mr. Langham, Mr. W. Johnson, Mr. Kinton, and Mr. I. F. Griffiths have been elected to the committee of the Hindley Cottage Hospital, Leicestershire.

Mr. Paget has been re-elected president of the Loughborough Hospital and Messrs. T. B. Jones and C. S. Taylor hon. auditors, while Parrs Bank, Ltd., have been appointed treasurers.

Mr. F. Crawley has been re-elected president of the Luton Bute Hospital, and Mr. E. Brown, Mr. W. Austin, Mr. F. W. Plummer, and Mr. G. Ordish have been re-elected governors.

Sir George Truscott, Bart., has been elected a vice-president of the London Homœopathic Hospital, and Mr. E. C. Brown and Colonel J. C. Tyler have been elected trustees. The two latter gentlemen were re-elected members of the Board of Management, with Mr. Archibald H. Campbell and Mr. William Willett.

